# Effects of flywheel resistance training on the running economy of young male well-trained distance runners

**DOI:** 10.3389/fphys.2022.1060640

**Published:** 2022-12-08

**Authors:** Yingying Weng, Haochong Liu, Tingting Ruan, Wenpu Yang, Hongwen Wei, Yixiong Cui, Indy Man Kit Ho, Qian Li

**Affiliations:** ^1^ School of Strength and Conditioning Training, Beijing Sport University, Beijing, China; ^2^ Cuiwei Primary School, Beijing, China; ^3^ Sports Coaching College, Beijing Sport University, Beijing, China; ^4^ Taizhou Science and Technology Vocational College, Taizhou, China; ^5^ AI Sports Engineering Lab, School of Sports Engineering, Beijing Sport University, Beijing, China; ^6^ School of Nursing and Health Studies, Hong Kong Metropolitan University, Hong Kong, China; ^7^ Asian Academy for Sports and Fitness Professionals, Hong Kong, China

**Keywords:** flywheel, resistance training, running economy, power, eccentric contraction

## Abstract

The study aimed to investigate the effect of flywheel accentuated eccentric loading (AEL) training on the running economy (RE) of young male well-trained distance runners. Twenty-two runners participated and were randomly assigned to the flywheel (FG, *n* = 12) and the control group (CG, *n* = 10). Traditional endurance training was performed in both groups three times a week for 6-week, while traditional resistance and flywheel AEL training was added to the CG and FG respectively. Subjects performed the incremental exercise test, squat jump, and countermovement jump (CMJ) before and after training. The results showed that 1) the RE at 65% of peak oxygen consumption (VO_2_peak), 75% VO_2_peak, and 85% VO_2_peak improved significantly after 6 weeks of training (*p* < 0.01, Effect size (ES) = 0.76; *p* < 0.01, ES = 1.04; *p* < 0.01, ES = 1.85) in FG, and the RE of 85% VO_2_peak in FG was significantly lower than CG (*p* < 0.05, ES = 0.30); 2) in post-training, both squat jump (*p* < 0.01, ES = 0.73) and CMJ (*p* < 0.01, ES = 1.15) performance, eccentric utilization ratio (*p* < 0.04, ES = 0.44), the rate of force development (RFD) of squat jump (*p* < 0.05, ES = 0.46), and CMJ_RFD_ (*p* < 0.01, ES = 0.66) were significantly improved in FG. And there are no significant differents in CG group because it was maintain training for our participants. Our findings showed that 1) flywheel AEL training improves the muscles’ explosive strength and other neuromuscular functions, and improves the athlete’s running economy under 65%, 75%, and 85% VO_2_peak, which potentially increases endurance performance. 2) Flywheel AEL training can improve the height, RFD, and the eccentric utilization ratio of squat jump and CMJ, and other lower limb elastic potential energy indicators of the young male, well-trained distance runners.

## 1 Introduction

Running economy (RE) and peak oxygen consumption (VO_2_peak) are considered two important physiological measures predicting the performance of trained distance runners. Given a submaximal running speed, the steady-state oxygen consumption is regarded as RE (Bassett and Howley, 2000) whereas, whereas VO_2_peak is the maximum amount of oxygen an individual can consume during exercise despite a further increase in workload over the period. Although VO_2_peak is a gold standard in assessing oxygen capacity and cardiovascular endurance, when compared with VO_2_peak, RE appears to be a better predictor of endurance performance (Noakes, 1988; Šuc et al., 2022), considering that anaerobic and neuromuscular properties both can affect endurance performance (Paavolainen et al., 1999; Nummela et al., 2006). It has been shown that strength training can effectively improve mechanical efficiency, muscular coordination, recruitment patterns of the motor unit, muscle and tendon stiffness, and ground contact time whereas all these can potentially enhance the RE and running performance (Sale, 1988).

In this regard, training strategies including low-resistance or high-resistance training, maximal strength training, explosive training, and plyometric exercises have been reported as effective ways to improve RE (Sale, 1988; Balsalobre-Fernández et al., 2016; Šuc et al., 2022). When compared to concurrent circuit training and endurance training, resistance training seems to be even more superior in enhancing RE values (Paavolainen et al., 1999; Millet et al., 2002; Taipale et al., 2010). In addition, the reutilization of elastic energy during the propulsive phase is more efficient when stretch-shortening cycle (SSC) performance (also known as reactive strength), and leg muscle stiffness of runners are improved after the resistance training program (Bubeck, 2010; Hernández-Davó et al., 2021; Šuc et al., 2022). In other words, runners can attain a certain running speed with substantially reduced energy consumption. As SSC is composed of an explosive concentric contraction (CON) immediately after rapid eccentric contraction (ECC), the effect of strength training with the predominant type of contraction (CON versus. ECC) on physiological adaptations and performance has been extensively studied. Interestingly, strength exercises using isolated ECC actions were reported to provide better force-using efficiency of lower limb muscle activation and metabolic cost than isolated CON action (Dudley et al., 1991). Similarly, recent studies also reported superior chronic training effect and adaptation in ECC training including a greater stimulation of Type IIx fibers (Duchateau and Baudry, 2014), and higher cortical activity (Tesch et al., 2004; Mendez-Villanueva et al., 2016). Moreover, eccentric training can effectively enhance muscle strength, induce morphological and structural adaptation of tendon tissue (Aagaard et al., 2000), significantly increase the cross-sectional area of muscles, cause higher mechanical tension in each active motor unit, greater pressure during muscle elongation, and a higher degree of exercise-induced muscle damage (EMID) (Toigo and Boutellier, 2006). Therefore, ECC training can greatly promote the hypertrophy of the fast twitch fibers, increase the proportion of the IIx muscle fibers, and such an increase in type IIx proportion was believed to be critical for speed and power output enhancement (Baroni et al., 2013).

Over the years, many different approaches have been adopted to enhance strength, including the use of free weights (Sagelv et al., 2020), weight stacks (Alkner and Bring, 2019), body weight (Fiorilli et al., 2020), and hydraulic machines (American College of Sports Medicine 2009) to perform strength training with concentric, isometric and eccentric actions. Although performing eccentric training on these strength training modalities is possible, when a sufficiently large overload on eccentric actions, also known as accentuated eccentric loading (AEL), is required, either a training partner or complicated equipment setup is needed. Moreover, these traditional training methods using constant external load were somewhat regarded as general strength exercises and not highly specific for athletes (Petré et al., 2018). Therefore, the versatile inertial training method using portable flywheel devices to provide AEL training has gained popularity in the past decades (Núñez et al., 2020). The iso-inertial devices, developed by Berg in 1994, transfers kinetic energy to the rotating disc during CON initiated by the athlete (Berg and Tesch, 1994). Additional ECC force is then required to slow the kinetic energy of the disk when the cable is maximally lengthened and therefore, the more force applied in CON or higher inertia (kg.m^2^) using more or larger disks is employed, the more ECC force is needed to increase the speed of the flywheel (Petré et al., 2018). Unlike traditional strength training equipment, flywheel device allows AEL training in multiplanar movements with the entire range of motion (Berg and Tesch, 1994). Moreover, the harness of the flywheel reduces the moment arm and helps in distributing the center of gravity during exercise. In this way, stress on the spine and the demand for technical skills for proper execution can be greatly reduced. With such biomechanical advantages, athletes can better focus on the AEL training on target muscles during exercises such as squats and lunges. In this regard, a growing body of evidence showed that the flywheel AEL method can clearly elicit training benefits including muscle mass (5–13% increase), maximal voluntary contraction (11–39%), strength in terms of 1 repetition maximum (1 RM) (12–25% enhancement), ECC force (21–90%), muscle power (10–33%), jumping ability (26–15%), running speed (2–10%), and muscle activity (up to 35%) (Petré et al., 2018).

Therefore, flywheel AEL is thought to be useful for a wide range of athletes to enhance sports performance. For instance, the physical capacities of soccer players (i.e., strength, power, jump, and direction changes) (Allen et al., 2021), lower-body strength and power qualities in male academy rugby players (Murton et al., 2021), and the countermovement jump (CMJ) performance of basketball players (Stojanović et al., 2021) were shown to be greatly improved using flywheel AEL. Nonetheless, studies regarding the benefits of flywheel AEL training on the RE performance for trained runners are limited. In this study, we investigated the effects of adding flywheel AEL to the endurance training program on the RE measures and jumping performance. The findings of this study provide empirical evidence that combined endurance training with flywheel AEL can elicit a clear additional performance enhancement for young well-trained distance runners when compared with the traditional strength training program.

## 2 Methods

### 2.1 Experimental approach to study

As a previous study has shown that the maximum adaptive capacity of RE occurs within 4–6 weeks of strength training (Turner et al., 2003), our study adopted parallel randomized controlled trials using a 6-week training program. Subjects were randomly assigned to an intervention or control group while pre-test and post-tests were performed to analyze the change in RE and jumping performance.

### 2.2 Subjects

Twenty-two young male long-distance runners were recruited and randomly divided into a flywheel group (FG, *n* = 12) and a control group (CG, *n* = 10) (Table 1). Beijing Sport University’s Research Ethics Committee has approved this study protocol (Registration number 2020008H). All procedures conformed to the Declaration of Helsinki. Informed consent was acquired prior to the experiment with all the benefits and potential risks associated with the study explained to participants. Participants in this study must meet the following inclusion criteria to minimize potential biases: 1) Participants were not injured in the past 6 months and; 2) Elite collegiate male long-distance runners with at least 5 years of training experience; 3) Participants could squat at least 1.5 times of their bodyweight.

**TABLE 1:** Physical characteristics of runners included in the analysis (baseline).

Group	Age (year)	Height (cm)	Body weight (kg)	Skeletal muscle (kg)	BMI (kg/m^2^)	Body fat (%)	Training age (year)	VO_2_peak (ml/min/kg)
FG (*n* = 12)	22.2 ± 2.6	177.6 ± 1.2	71.3 ± 7.4	36.9 ± 4.0	22.5 ± 1.5	10.1 ± 3.9	5.2 ± 1.6	58.3 ± 3.4
CG (*n* = 10)	21.4 ± 2.5	178.8 ± 2.2	71.9 ± 5.7	37.0 ± 2.9	22.6 ± 1.5	10.7 ± 3.8	4.5 ± 2.0	57.8 ± 3.3

### 2.3 Training program and prescription

**FIGURE 1:**
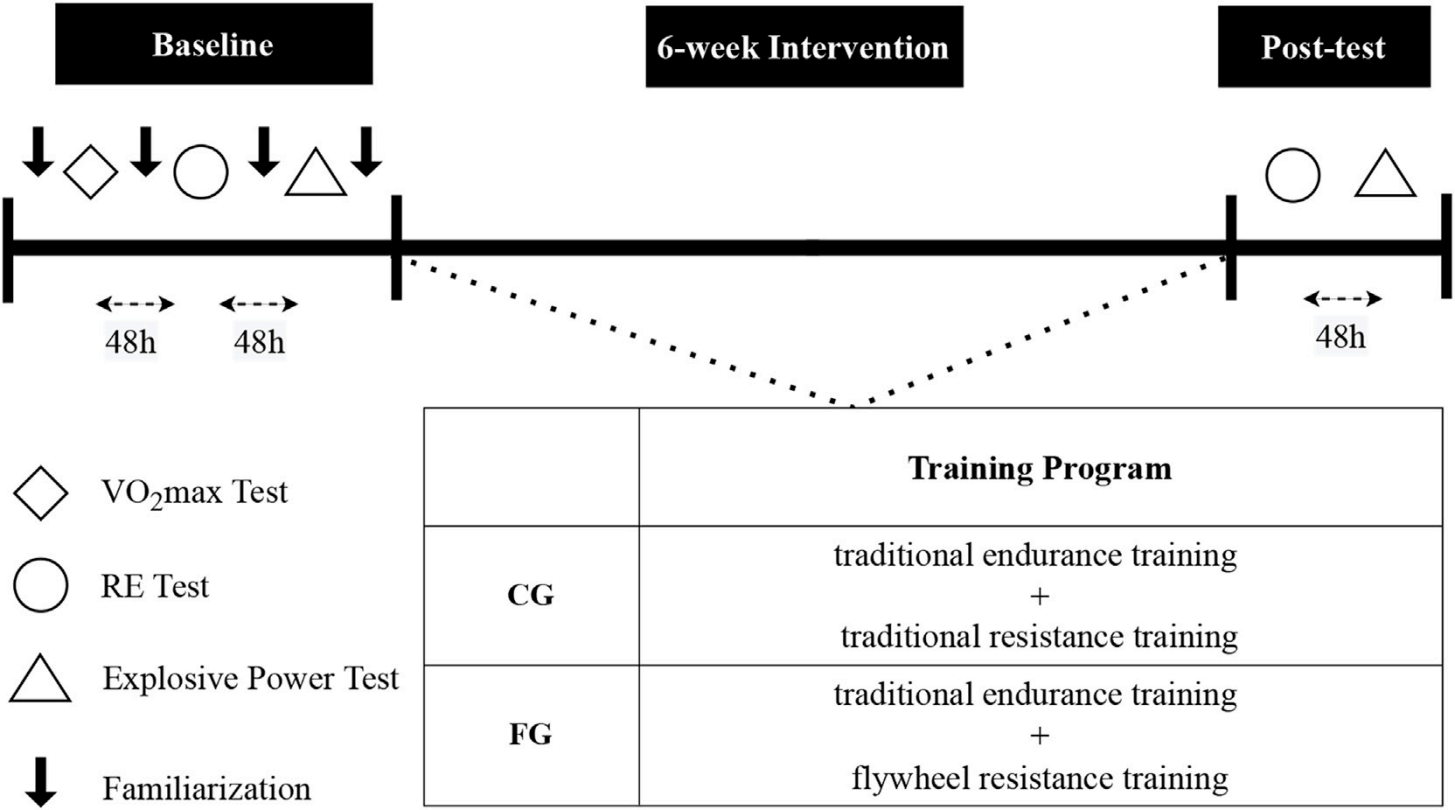
Schematic diagram of the experimental process.

The flow of this study was illustrated in Figure 1. All participants received a 2-week familiarization period to fully understand the proper techniques and get used to both flywheels AEL and resistance training before the experiment. Training sessions for the FG and CG groups were conducted three times a week, with a recovery period of at least 24 h between sessions. During the 6-week training, the CG group performed a 10-km endurance running followed by a traditional strength training program with 6–8 h of rest given between the two programs. Each session the strength training was comprised of 4 sets of barbell squat exercises (6 reps) using 85% of 1 repetition maximum (RM). Participants were asked to squat until their thighs were parallel to the floor and push as hard and fast as possible (Sagelv et al., 2020; Spudić et al., 2020). A 3-min rest was given between sets of an exercise to allow complete recovery. The FG group performed a 10-km endurance training similar to the CG group. For the strength part, participants in FG condition performed a flywheel squat training program with 4 sets of 7 reps using an inertial load of 0.06 kg m^2^ and a 3-min inter-set rest (Table 2) (Tous-Fajardo et al., 2006; González-Badillo and Marques, 2010; Tesch et al., 2017). During the flywheel training, all relevant parameters were monitored in real-time according to the participant’s performance for timely adjustments to ensure centrifugal overload. Both groups only performed the assigned resistance exercise and did not do other additional training.

**TABLE 2:** Schedule of different training programs.

Training types	Training programs
Traditional strength training	Squat (85% 1RM), 6 reps × 4 sets, 3 min interval, 3 times a week
Flywheel resistant training	Squat with maximum efforts (flywheel inertia load of 0.06 kg·m^2^), 7 reps × 4 sets, 3 min interval, 3 times a week
Endurance training	10 km continuous endurance run, 3 times a week

### 2.4 Test program

The before and after tests consisted of the VO_2_peak test, running economy index analysis at speeds of 65%,75%,85% of VO_2_peak, and explosive power test. Each test was at least 48 h apart to eliminate fatigue or any carryover effect. Participants refrained from any physical activity and intake of caffeine-containing food or beverage before or in between tests. All subjects completed baseline tests before starting the 6-week training program, and subjects repeated the same testing procedures thereafter in the same order.

#### 2.4.1 Incremental test to exhaustion

Peak oxygen consumption and heart rate were determined during incremental maximum running tests on the treadmill, by respiratory analysis of oxygen consumption and carbon dioxide production (CORTEX Biophysik GmbH, Germany). Followed by a 10-min standardized warm-up, coming to the test protocol: 8 km/h—16 km/h in increments of 1 km/h per minute, the treadmill (h/p/cosmos Mercury 4.0, Germany) started increasing the incline by 1.5% per minute after reaching 16 km/h. The experiment was terminated when three of the criteria shown below occurred during the experiment (Beaver et al., 1986). The VO_2_peak value with the maximum oxygen uptake and the maximum heart rate was recorded.

a. RQ respiratory quotient >1.15.
b. Heart rate >180 bpm.
c. Subjects reached the force exhaustion condition (Borg RPE 6–20 scale).
d. Oxygen uptake plateaus (VO_2_ variation range ≤150 ml/min) were observed.

#### 2.4.2 Running economy test

We calculated RE by measuring submaximal oxygen consumption while running on a treadmill (Barnes and Kilding, 2015). The treadmill runs were performed for 5 min at speeds of 65%, 75%, and 85% of VO_2_peak respectively after a standardized warm-up period (3 min at a slow pace) on the same day. Heart rates were monitored with the Cortex Gas Metabolic Analysis System (Metalyzer 3B, Cortex Biophysik, Leipzig, Germany). We defined the RE as the mean VO_2_ collected at each speed at the last minute. To facilitate comparison and analysis, the intensity of constant load tests before and after the experiment was based on the velocity corresponding to the percentage of VO_2_ in the baseline.

#### 2.4.3 Explosive power test

For vertical jump performance, all subjects performed a squat jump (SJ) and CMJ, and both of these were measured by a 3D force platform (Kistler 9281CA, Switzerland) (Bosco et al., 1983). Calculating the jumping height using a kinematics equation was done based on the flight time. The Kistler dynamometer recorded and output flight time (T) and force (F). Eccentric utilization ratio (EUR) = CMJ/SJ, differences in EUR ratio were compared before and after strength training. The rate of force development (RFD) = △F/△t was also calculated.

### 2.5 Statistical analyses

All experimental data were analyzed by IBM SPSS statistical software (version 23.0, IBM, Chicago, IL, United States). Normality tests of dependent variables were verified and all passed with Skewness-Kurtosis tests. Descriptive statistics were presented using the mean ± standard deviation ( $\bar{x}$ ± s). A lower RE value indicated better running efficiency and reduced energy consumption. Independent sample t-tests were used to examine the physical characteristics of runners included in the analysis (baseline). The difference between groups was using two-way repeated measures ANOVA (Time * Group), while the difference among different VO2peak levels was using three-way repeated measures ANOVA (Time * Group * RE). We examined their interactions and conducted post-hoc analysis when they were significant. And these statistics presented effect size (ES) as a standardized mean difference (Cohen's d) between two groups. The classification of effect size (ES) such as small = 0.2, moderate = 0.5, large = 0.8 (Cohen, 1988).

## 3 Results

### 3.1 RE performance

**FIGURE 2:**
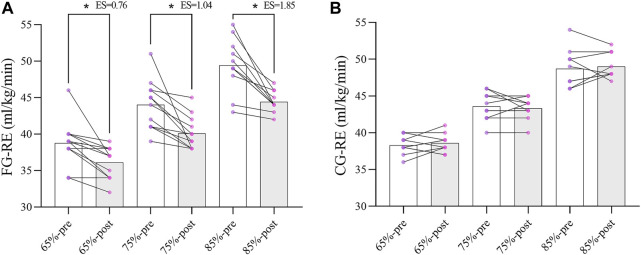
Change in RE parameters between two groups (* *p* < 0.05; *** *p* < 0.001).

The results of ANOVA models on FG showed the significant effects of different VO_2_peak, that is, compared to the baseline, during 65% VO_2_peak (F_(1,11)_ = 11.22, *p* = 0.006, ES = 0.76), 75% VO_2_peak (F_(1,11)_ = 22.64, *p* = 0.001, ES = 1.04), and 85% VO_2_peak (F_(1,11)_ = 42.63, *p* = 0.000, ES = 1.85) participants demonstrated significantly lower RE values in the post-tests (Figure 2). There was no significant difference in the RE value between pre-and post-test of CG at different levels of VO_2_peak. Regarding the differences between groups, there was no significant difference in RE performance between FG and CG groups on the pretest, but the two groups differed significantly on the posttest (F_(1,20)_ = 5.26, *p* = 0.033), with the FG group (40.22 ± 1.70) performing significantly lower (or better) than CG group (42.30 ± 2.53).

Simple effects analysis of third-order interactions for different levels of RE performance, only in 85% VO_2_peak, in the post-test, the RE performance of the FG group (44.42 ± 1.38) was significantly lower than that of the CG group (46.9 ± 2.47) (F_(1,20)_ = 8.87, *p* = 0.007). There was no significant difference in other conditions (Table 3).

**TABLE 3:** Three-way repeated measures ANOVA for subjects in different groups and test situations at different levels of running intensities.

Source of variation	*F*	*p*	*η*_p_^2^	Observed power
Time	17.61	0.00	0.47	0.98
Time × Group	25.01	0.00	0.56	1.00
Intensity	277.78	0.00	0.93	1.00
Intensity × Group	0.00	0.99	0.00	0.05
Time × Intensity	11.31	0.00	0.36	0.99
Time × Intensity × Group	6.17	0.01	0.24	0.87

### 3.2 Jump performance and kinetic parameters

**FIGURE 3:**
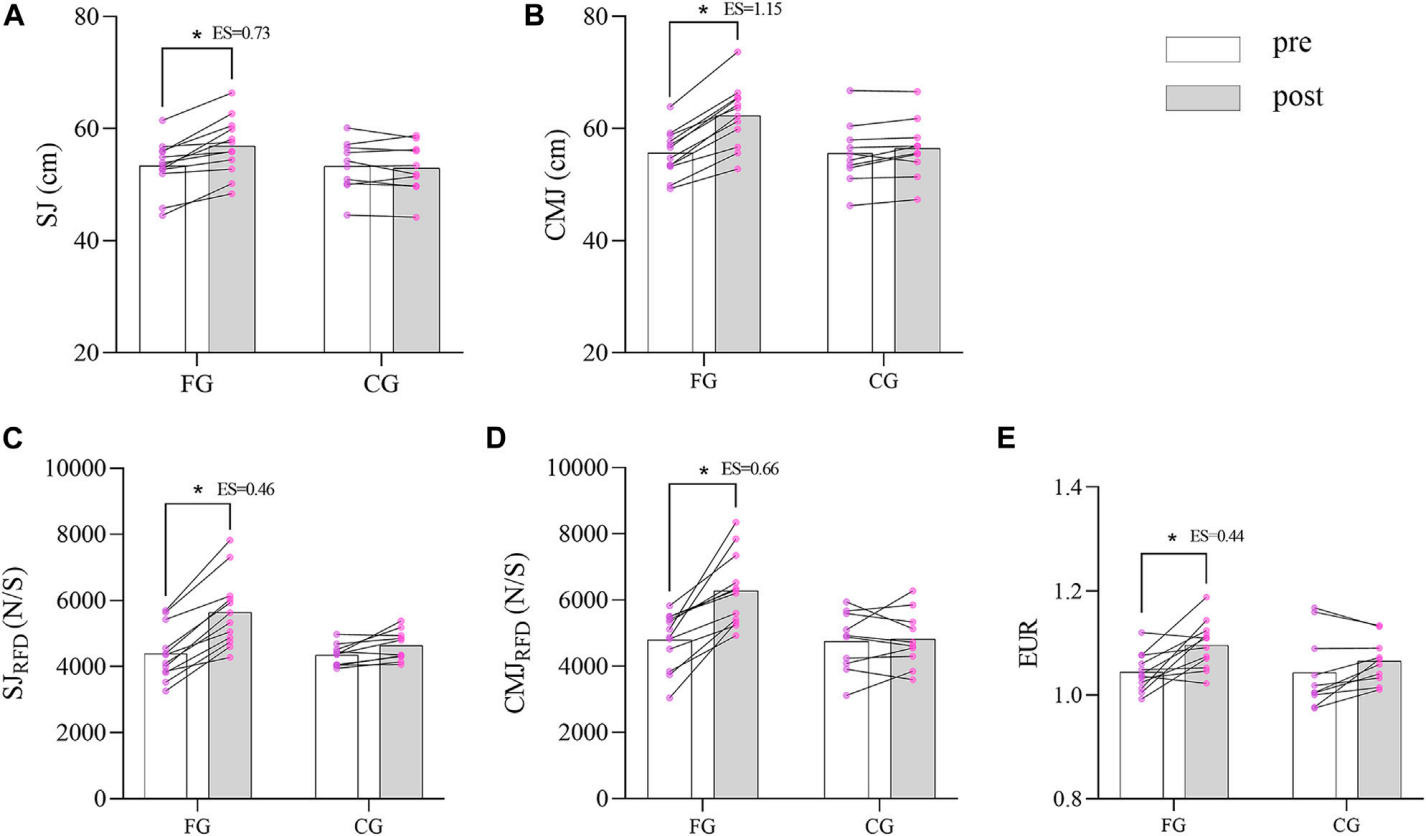
Change in Kinematic parameters between two groups (* *p* < 0.05; *** *p* < 0.001).

When compared the pre-and post-tests, significant improvements were observed in SJ (t_(11)_ = 5.25, *p* < 0.001, ES = 0.73), CMJ (t_(11)_ = 9.77, *p* < 0.001, ES = 1.15), EUR (t_(11)_ = 2.32, *p* = 0.04, ES = 0.44), SJ_RFD_ (t_(11)_ = 3.98, *p* = 0.013, ES = 0.46) and CMJ_RFD_ (t_(11)_ = 3.98, *p* = 0.002, ES = 0.66) only in FG. No significant difference was found in the CG after the 6-week traditional training program (Figure 3).

## 4 Discussion

This study aimed to compare the effect of flywheel AEL and traditional resistance training on the RE and jumping performance of young male distance runners. Our results indicated that AEL training using a flywheel device on top of regular endurance training could elicit significant performance benefits on both RE and lower limb explosive power. Interestingly, the such performance gain was not observed in the CG group using combined training with endurance and traditional strength methods.

To enhance RE performance, neuromuscular characteristics such as muscle force production, eccentric capacity, elastic energy utilization, and leg stiffness play an important role (Li et al., 2021). Resistance exercises based on multi-joint and free-weight forms of training (Blagrove et al., 2018), thus improve neuromuscular function, increase the number of motor units recruited, and enhance both intramuscular and intermuscular coordination, increase muscle strength and contraction velocity (Folland and Williams, 2007), and reduce the energy consumption at the same running speed (Millet et al., 2002; Spurrs et al., 2003).

Our results have shown clear and substantial improvements in RE at different running intensities (65%–85% of VO_2_peak) in the FG group after receiving flywheel resistance training but not in the CG group. Although previous studies have reported great potential benefits of adding resistance training to the usual running routine on RE and strength enhancement for runners (Sale, 1988; Paavolainen et al., 1999; Millet et al., 2002; Taipale et al., 2010; Balsalobre-Fernández et al., 2016; Šuc et al., 2022), our subjects were already well-trained in strength training (capable squatting at least 1.5 times of their bodyweight) and therefore, the such additional performance gain was not observed in CG group. In this regard, flywheel training seemed to be capable of further enhancing running and strength performance for those runners with good strength training experience.

In addition, the benefits of RE enhancement in the FG group were more prominent in our running test using a higher speed or intensity (85% VO_2_peak: 9.8% RE improvement and ES = 1.87, 65% VO_2_peak: 6.6% and ES = 0.76). It is worth noting that from the biomechanical perspective, slow and fast running can demonstrate different characteristics. Previous studies adopted the spring-mass model to explain the bounce of the body during each step of running. The “spring” of the muscle-tendon units should be compressed (i.e., stretching of the tissues) and absorb the kinetic energy during the landing phase while it extends to take-off (i.e., shortening of the muscle-tendon units). In this regard, it was assumed that the height and velocity of the center of mass at landing are equivalent to the take-off phase (symmetric). Nevertheless, previous studies split the entire running cycle into 4 distinctive moments including the downward acceleration and deceleration, and the upward acceleration and deceleration, and they found that the landing-take-off processes are asymmetric in nature (Cavagna, 2006; Cavagna et al., 2008; da Rosa et al., 2019). In this regard, such landing-take-off asymmetry provides the support of the body from the vertical vector being larger during the lifting than the downward phase. With the aid of gravity, the maximum downward kinetic energy in the running step allows a high-velocity stretch of the muscle-tendon units while a longer and slower push is executed during the upward-lifting process. Such an asymmetry was thought to be important to exert greater average force during landing (stretching) than that developed during the take-off (shortening) and these allow maximal efficiency of positive work production in ramp SSC, especially during the slow and moderate running speeds. Interestingly, it was also shown that such an elastic bouncing feature in the running cycle is dependent on the performance level of runners. Well-trained runners demonstrate the spring-mass system oscillating faster with larger vertical amplitude and hence, leading to a larger stride length.

From the metabolic perspective, energy cost can be regarded as the metabolic energy demand for moving 1 kg of body mass over 1 m (J kg^−1^m^−1^), and interestingly, it seems that the energy cost of running is not necessarily affected by the running speed (Peyré-Tartaruga et al., 2021). Therefore, we assume that our subjects were required to overcome the same energy cost (4 J kg^−1^m^−1^) to drive their body forward despite the different running intensities. Meanwhile, the total energy required in running should include the gravitational potential energy, forward kinetic energy, mediolateral kinetic energy, and vertical kinetic energy. Runners have to better recycle mechanical work by storing and releasing more elastic energy or generating additional mechanical power to produce a larger stride length (by more forward and upward mechanical works) or higher stride frequency to achieve a faster running pace. When running at a higher intensity (i.e., 85% VO_2_peak), apparent efficiency (the ratio between mechanical work and energy cost, or defined as the efficiency of the positive work production by the musculotendinous units) increases (Peyré-Tartaruga et al., 2021). Therefore, the demand for power production from the muscle-tendon units during high-intensity running (i.e., 85% VO_2_peak) should be higher than that of slow running (i.e., 65% VO_2_peak). Furthermore, as running at a higher level of VO_2_ is more demanding on the anaerobic energy system, the recruitment of type II fibers, and power production for propelling the body forward, the use of flywheel resistance training was thought to have important contributions in this regard. A recent meta-analysis indicated that flywheel AEL training improves CMJ performance more effectively than traditional resistance training methods (Maroto-Izquierdo et al., 2017). Similarly, another recent systematic review also reported that chronic flywheel resistance training appears to be more effective than traditional strength training in enhancing the capabilities in the change of direction in male athletes. Nevertheless, prior studies did not examine or highlight the change in EUR, RFD, and SJ.

Our study has demonstrated significant improvements in both EUR, SJ_RFD_, and CMJ_RFD_ in FG groups trained with flywheels. Elastic energy utilization involving storage and return of mechanical energy greatly contributes to reduced energy consumption during running (Sasaki and Neptune, 2006). The most important neuromuscular factors related to elastic energy utilization are stretch-shortening cycle (SSC) (Vogt and Hoppeler, 2014), leg stiffness (Moore, 2016), step frequency (Cavagna et al., 2008) and stretch–recoil of the tendons (e.g., a higher frequency and larger vertical amplitude result in a greater stride length) (Cavagna, 2006; da Rosa et al., 2019). EUR has been demonstrated to be a reliable indicator of SSC performance across a wide range of sports and different training stages, and it has been endorsed by many coaches and researchers (Haff G and Molinari, 2010; Kipp et al., 2021). Runners with a better ability to use the SSC have approximately 50% greater force during the push-off phase after eccentric contraction and have a reduced running metabolic cost (Vogt and Hoppeler, 2014). As a result, an increase in EUR indicates an increase in elastic energy storage and utilization during the SSC cycle, which contributes to RE enhancement. Moreover, the positive change in power measures including SJ and CMJ indicated better potential in producing upward and forward propulsive force during the acceleration of a running movement. Flywheel AEL training with increased loading during the resistance phase may improve the mechanical properties of skeletal muscle, RFD, and the ability of active muscle to utilize elastic energy during the SSC cycle. Moreover, previous studies have proposed the potential benefit of using AEL in increasing the ratio of type IIx fiber and hence the power production (Walker et al., 2020). With better elastic energy utilization and power production capabilities, it is believed to improve both the reactive strength and efficiency of muscle work. Subjects could therefore require less time to decelerate and re-accelerate per step. By reducing the effort and time of foot landing during the supporting phase (amortization phase of SSC), it improved transition time and reduced the cost of energy.

The present study has some limitations. Since only male trained runners were recruited, our findings may not be able to generalize to other populations such as female runners, or runners with different levels. Further studies, and on with larger sample sizes in different populations are warranted. To the author’s knowledge, only one study combines flywheel AEL training and aerobic endurance programs, which shows improvements in RE and neuromuscular adaptation (Festa et al., 2019). The variables of an optimum AEL training program to produce the largest RE and running enhancement are yet to be identified. Future studies comparing the effects of various training loads, frequencies, and formats on RE performance are warranted. In our study, we did not observe a change in running posture and muscle stiffness after flywheel AEL training. Therefore, to what extent these qualities contributed to the performance gain is not well understood. Meanwhile, we did not observe the relationship between RE and biomechanics, like the alterations to running technique (e.g., stride frequency, stride length) (Tartaruga et al., 2012) and terrain feature (e.g., level, hill) (Melo et al., 2022). Therefore, it is inconclusive regarding the contribution and impact induced by the change of biomechanics or running techniques after AEL training on the observable RE improvement. Meanwhile the current study did not examine the effect of combining AEL and traditional strength training on RE and other performance indicators. Further studies in this regard are warranted to understand if AEL training alone or AEL on top of traditional resistance training can yield additional performance gain. Furthermore, despite the same number of exercise and set, the total training volume, training load and exercise tempo in our FG and CG groups were slightly different due to the nature of the resistance training method. Further studies using equivalent training load and volume between AEL and traditional strength training are recommended. In addition, we included only one control group that received traditional strength training. It is worth including a blank control group to figure out the single-type training effect of flywheel training in the future.

## 5 Conclusion

Flywheel AEL training improved the lower limb explosive strength and jump performance including EUR, SJ, CMJ, and RFD of young male well-trained distance runners. Meanwhile, it also improved the athlete’s running economy under 65%, 75%, and 85% VO_2_peak, which potentially increased endurance performance. Moreover, the AEL training can be achieved by portable flywheel device whereas traditional high intensity strength training (e.g., Six RM) requires massive equipment including barbell, weight plates and rack. Apparently, flywheel AEL training with comparable exercise volume potentially provide superior training benefits to the traditional resistance training. Therefore, running or strength coaches and athletes who cannot access or carry strength training facilities should consider adding flywheel AEL training into their usual endurance for acquiring performance gain (American College of Sports Medicine, 2009).

## Data Availability

The original contributions presented in the study are included in the article/supplementary material, further inquiries can be directed to the corresponding author.
